# Periareolar minimally invasive approach for cardiac surgery: a case series and description of technique with a review of literature

**DOI:** 10.1186/s13019-024-02948-9

**Published:** 2024-08-01

**Authors:** Hatan Mortada, Abdulaziz Alsuhaim, Nasser Alkhamees, Omar Fouda Neel

**Affiliations:** 1 Division of Plastic Surgery, Department of Surgery, King Saud University Medical City, King Saud University, Riyadh, Saudi Arabia; 2 Department of Plastic Surgery & Burn Unit, King Saud Medical City, Riyadh, Saudi Arabia; 3 College of Medicine, King Saud University, Riyadh, Saudi Arabia; 4 Division of Cardiac Surgery, King Fahad Cardiac Center, King Saud University Medical City, King Saud University, Riyadh, Saudi Arabia; 5 Division of Plastic Surgery, Department of Surgery, King Saud University Medical City, King Saud University, Riyadh, Saudi Arabia Saudi Arabia; 6 Division of Plastic Surgery, Department of Surgery, McGill University, Montreal, Canada

**Keywords:** Cardiac surgery, Minimally invasive, Periareolar approach, Patient reported outcome, Surgical technique

## Abstract

**Background:**

Minimally invasive cardiac surgery (MICS) has garnered significant attention for its potential benefits, including decreased surgical trauma, accelerated recovery, and improved aesthetic outcomes. This case series aims to elucidate the technical aspects and assess the aesthetic, functional, and quality of life outcomes associated with the utilization of a periareolar incision approach in female patients undergoing cardiac surgery.

**Methods:**

The periareolar MICS technique, performed with or without high-definition (HD) 3D endoscopic visualization, limited rib-spreading, and a periareolar incision spanning the 3 to 9 o’clock positions, was employed. We present a case series encompassing five female patients who underwent various cardiac procedures for different pathologies using this approach.

**Results:**

No intraoperative complications occurred, and all patients experienced uneventful postoperative recoveries. The periareolar approach resulted in well-healed incisions with minimal scaring, preserving breast contour and yielding satisfactory cosmetic outcomes. Patients reported negligible pain levels and expressed contentment with the scar appearance.

**Conclusion:**

The periareolar incision technique in MICS represents an efficacious approach characterized by favorable aesthetic outcomes and enhanced patient experience. Further investigations are warranted to compare different MICS approaches with respect to pain management and their impact on quality-of-life domains.

## Introduction

Minimally invasive procedures offer a range of potential benefits, including reduced surgical trauma, decreased pain, minimized blood loss, shorter hospital stays, faster recovery, improved cosmetic outcomes, and enhanced patient satisfaction [1–3]. Within specialized centers, minimally invasive mitral valve repair (MIMVr) or replacement (MIMVR) has become a standard procedure [4]. In the context of cardiac surgery, optimizing both aesthetic and functional aspects is of utmost importance. Patients undergoing MIMVr, for instance, experience earlier resumption of activities, reduced wound infections, decreased trauma, and expedited recovery [5]. Therefore, a comprehensive understanding of different minimally invasive surgical approaches can significantly enhance the overall patient experience. In the field of plastic and reconstructive surgery, zigzag transareolar approaches have been employed to achieve a close approximation of the nipple and improve exposure. However, concerns persist regarding scar appearance and the potential risks of ductal injury and capsular contracture [6]. From a cosmetic standpoint, it is crucial to investigate whether the periareolar approach technique offers superior aesthetic outcomes in terms of breast shape, postoperative scarring, and satisfaction when compared to other minimally invasive cardiac surgery (MICS) approaches [7]. Although Durdu et al. have conducted a comparison between minimally invasive and conventional surgeries, limited research exists on the comparison of different types of MICS approaches in relation to postoperative pain and quality of life [8]. Therefore, the objective of this study is to evaluate the aesthetic and functional outcomes, as well as the impact on quality of life, in patients who have undergone cardiac surgery utilizing the periareolar incision technique at our institution. Moreover, we have conducted a thorough review of the literature, summarizing, and presenting the findings in a concise and informative manner, including a review of relevant studies in a dedicated literature review table.

## Surgical technique

All patients underwent periareolar MICS +/- HD 3D endoscopic visualization with minimal rib-spreading. A soft tissue retractor with a maximum diameter of 4 cm is utilized, along with a rib spreader when necessary for further exposure. During the procedure, patients are positioned in a 30-degree left lateral decubitus position. In all cases, transesophageal echocardiography (TEE) was performed to confirm preoperative diagnosis and evaluate postoperative end results. Following appropriate sterile preparation and draping, 5000 units of heparin are given to target an ACT more the 200 msec. Our Cardio-Pulmonary bypass (CPB) perfusion technique consists of femoro-femoral cannulation combined with internal jugular cannulation. The right common femoral vessels are exposed through a 2 cm oblique groin incision. Under ultrasound and TEE guidance, Seldinger technique is used to cannulate the right internal jugular vein with a 17 F cannula, while the exposed right common femoral vein is cannulated with a 25 F multistage cannula. Arterial cannulation is obtained through sewing an 8 mm Hemashield graft to the right common femoral artery. At this point, the plastic surgery team conducts a preoperative marking for a classic periareolar breast augmentation technique, with an incision ranging from 3 o’clock to 9 o’clock. Size of the incision differs from one patient to another depending on the areolar size. Dissection is performed in an inferior fashion, leaving a 2 cm thick S flap extending down to the prepectoral pocket. Dissection above the pectoralis muscle is carried out at the level of the 4th intercostal space, similar to prepectoral breast augmentation techniques. If a breast implant is present, the implant will be removed to expose the thoracic wall.

A right mini thoracotomy is performed in the fourth intercostal space, and a 2 − 0 ethibond pledgeted suture is used to retract the diaphragm caudally. The pericardium is opened 3 cm above the phrenic nerve then suspended laterally. Two pledgeted 3 − 0 prolene sutures are placed on the aorta below the planned cross clamp level for the cardioplegia cannula. Once the cardioplegia cannula is inserted, a transthoracic “Chitwood” aortic cross clamp is applied. Custodiol cardioplegia (1.5 L) is infused in an antegrade fashion to induce cardiac arrest during diastole. Patients body temperature is cooled down to 32 degrees celsius as a protective strategy for the brain and other organs during CPB. The left atrium is accessed through sondergaard’s groove while the right atrium is accessed through an oblique right atrial incision. In this study, the scar outcome at different stages in all patients was carefully assessed. All patients included in the study provided their consent to have their cases reported, and corresponding figures showcasing the scar progression were attached to support the findings. Patients were considered for inclusion in this study if they were female, had an indication for cardiac surgery, and expressed a preference for a minimally invasive approach with an emphasis on aesthetic outcomes. Exclusion criteria included male gender, inability to provide informed consent, and the presence of contraindications to minimally invasive surgery, such as severe pulmonary hypertension or previous right thoracic surgery. Patients with a history of breast surgery or implants were not excluded from this study.

### Case study − 1

A 29-year-old female patient with a history of mitral valve prolapse with severe mitral valve regurgitation, as well as bilateral prepectoral breast augmentation, presented for minimally invasive mitral valve repair utilizing a periareolar incision approach with right-sided implant revision. The patient had an American Society of Anesthesiologists (ASA) classification of 3. Prior to the cardiac procedure, the plastic surgery team performed a periareolar incision on the right breast, extending from 3 to 9 o’clock, exposing the chest wall. Subsequently, the cardiac surgery team proceeded with the mitral valve repair using a bileaflet neochordae implantation technique and a slightly oversized annuloplasty ring. During the procedure, the plastic surgery team conducted an implant revision and closed the incision in a standard multilayer fashion. The estimated cross clamp time was 103 min, and no intraoperative complications occurred.

Following the surgery, the patient was admitted under the care of the cardiac surgery team for a five-day duration, including one day in the intensive care unit (ICU). The patient’s postoperative course was uneventful, apart from experiencing multiple episodes of delirium. Regarding the breast augmentation aspect, upon admission to the hospital, the patient reported no pain, redness, tenderness, hematoma, discharge, or signs of infection at the incision site. Subsequent follow-up visits at the clinic revealed a well-healed, flat scar with preserved breast shape. The scar size measured approximately 3 cm, without any noticeable hypo/hyperpigmentation changes. The patient expressed satisfaction with the augmentation results and scar outcome.

The patient reported temporary numbness around the areola and nipple for the initial two weeks following the surgery, which is a known and expected complaint associated with the periareolar incision technique, as documented in the literature. It is important to note that an areola diameter smaller than 3 cm can pose challenges in exposing cardiac structures during the surgery, sometimes requiring additional efforts, and potentially resulting in extensions beyond the areolar region, thereby increasing the risk of numbness in that area. However, in this particular case, the patient did not experience any complications related to the surgical approach. Additionally, the patient mentioned a small breast lump on the lower aspect of the breast, but subsequent radiological studies confirmed benign findings.

### Case study − 2

A 17-year-old female patient was diagnosed with a superior sinus venosus atrial septal defect (SV-ASD) with partial anomalous pulmonary venous drainage (PAPVD) since birth. She had no other underlying medical conditions and was classified as ASA 3 by the anesthesia team. The anatomy of her ASD and associated anomalies necessitated surgical intervention. The plastic surgery team performed a periareolar incision encircling the areola, extending from 3 to 9 o’clock, to gain access to the chest wall and heart. The cardiac surgery team proceeded with the repair of the SV-ASD and the PAPVD using a two patch (bovine pericardium) technique, where a baffle was created to direct the drainage of the right superior and middle anomalous pulmonary veins to the left atrium and a second patch to enlarge the cavoatrial junction to prevent SVC narrowing. The total estimated cross clamp time was 164 min, and the operation proceeded smoothly without encountering any complications.

Following the procedure, the patient was initially admitted to the Intensive Care Unit (ICU) and subsequently transferred to the ward under the care of the cardiac surgery team and discharged home on the 4th day postop. Throughout this period, there were no indications of pain, redness, swelling, discharge, or signs of infection at the incision site. The wound was diligently maintained, with regular assessments of its integrity, cleaning, and dressing performed by the plastic surgery team. During follow-up visits at the clinic, the patient did not report any concerns regarding the periareolar incision scar. No instances of numbness, pain, or discoloration were reported by the patient. In fact, the scar exhibited clarity and a flat appearance, characterized by well-defined borders. The patient expressed satisfaction with the overall outcome of the scar. The size of the scar measured approximately 2.8 cm, with minor hypopigmentation changes noted.

### Case study − 3

A 35-year-old female patient was diagnosed with Left Atrial Myxoma after presenting with a headache and blurry vision. Aside from this condition, she had no other underlying medical issues and was classified as ASA 3 by the Anesthesia team. A transesophageal echocardiogram showed that tumor was located at the cranial aspect of the left atrium at the entrance of the right superior pulmonary vein. Surgical resection of the tumor was planned for the patient. The surgical approach involved a periareolar incision performed by the plastic surgery team, extending from 3 to 9 o’clock. This incision provided adequate exposure for the cardiac surgery team to perform an en Bloc resection of the myxoma. Following the successful resection, the plastic surgery team meticulously closed the incision. The total estimated cross clamp time was 92 min. The patient was admitted to the Intensive Care Unit (ICU) initially and subsequently transferred to the ward, she was discharged home on the 6th day postop. Throughout her hospitalization, the plastic surgery team consistently monitored the wound, which exhibited no signs of pain, redness, swelling, discharge, or infection. The wound was diligently cleaned and appropriately dressed.

During follow-up visits at the clinic, the scar was found to be clean with well-defined margins, flat, and mildly hyperpigmented. The patient expressed satisfaction with the outcome of the scar, reporting no history of color changes or raised areas at the incision site. However, she did experience numbness at the site of the incision for a duration of 1 month following the surgery.

### Case study − 4

A 42-year-old female patient was diagnosed with a large size secundum atrial septal defect (ASD) with a deficient inferior rim. She had no other concurrent medical conditions and was assessed by the anesthesia team, who classified her as ASA 3. The surgical approach utilized in her operation was consistent with the previously mentioned cases, involving a periareolar incision performed by the plastic surgery team, extending from 3 to 9 o’clock. The ASD was successfully patched using bovine pericardium, with no complications encountered during the procedure. The total estimated cross clamp time was 100 min.

**Fig. 1:**
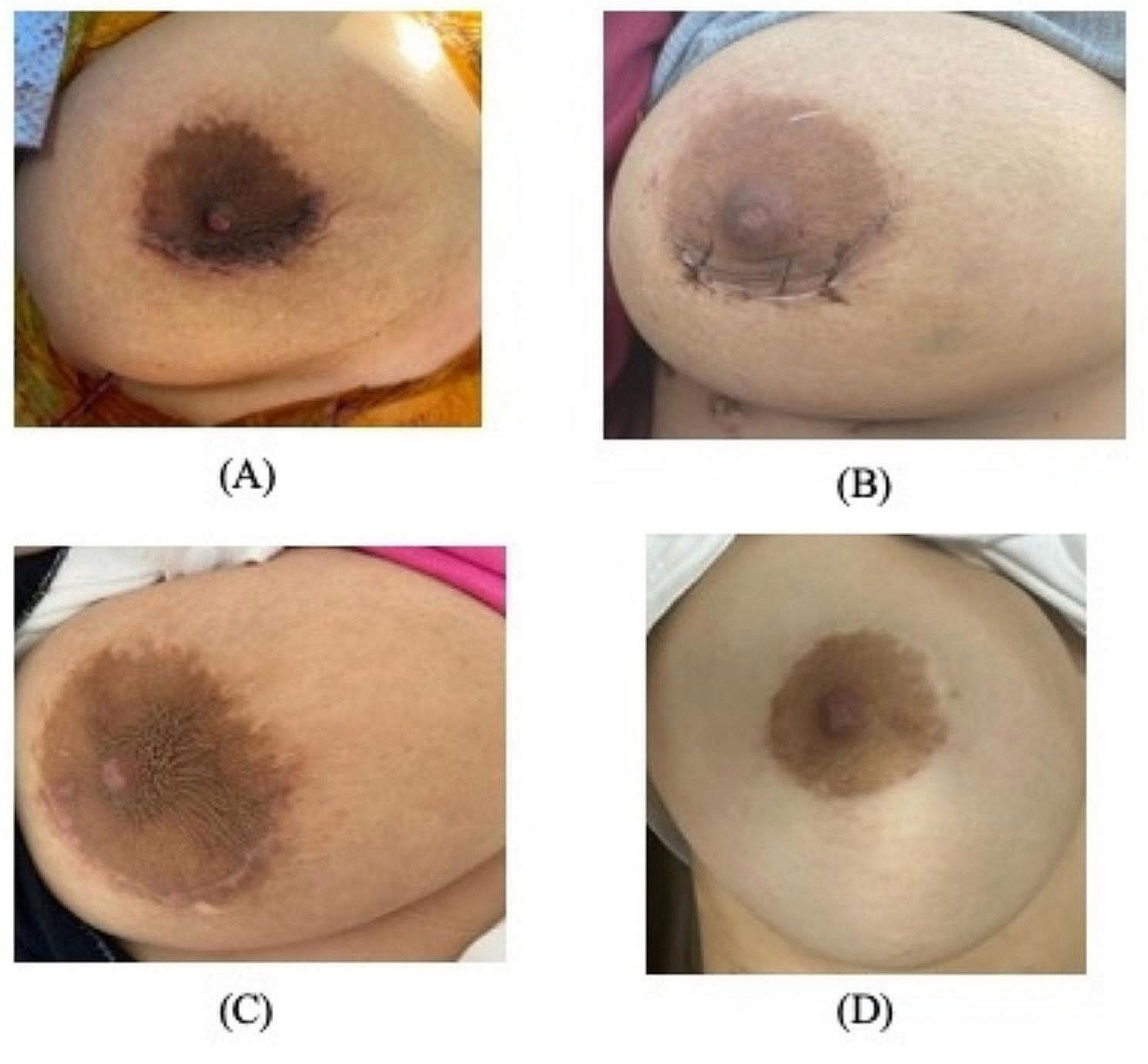
Scar Outcome at Different Stages in Different Patients. (**A**) Immediate postoperative scar after skin closure. (**B**) Scar appearance at 10 days after closure. (**C**) Scar appearance at 2 months after surgery. (**D**) Scar appearance at 4 months after surgery, where the scar is nearly invisible

Following the surgery, the patient was admitted to the ICU and subsequently transferred to the ward and was discharged home on the 5th day postop. Throughout her hospital stay, the plastic surgery team consistently monitored and assessed the wound, which remained clean and exhibited no indications of pain, redness, swelling, discharge, or infection. During the patient’s follow-up visit at the clinic, she expressed satisfaction with the outcome of the scar. The scar appeared clean, flat, with well-defined margins. Overall, there was minimal pigmentation, except for a slight hypopigmentation noted at the 5 o’clock position. However, the patient conveyed contentment and satisfaction with the overall result (Fig. 1).

### Case study – 5

A 57-year old female patient was diagnosed with secundum ASD and cor triatriatum sinister. She had no other concurrent medical conditions and was assessed by the anesthesia team and classified as ASA 3. The surgical approach utilized in her operation was consistent with the previously mentioned cases, involving a periareolar incision performed by the plastic surgery team, extending from 3 to 9 o’clock. However, in this case a cor triatriatum sinister was present. Upon identification, resection of the fibromuscular membrane with complete visualization of the left atrium including the mitral valve and pulmonary veins was achieved. The ASD was patched utilizing bovine pericardium with no complications encountered during the procedure. The total estimated cross clamp time was 173 min. The patient was admitted to the ICU after the surgery and she was subsequently transferred to the ward. The wound was assessed and monitored daily by the plastic surgery team. On the 6th day postop, the patient was discharged home with no cardiac or wound complications. A comprehensive summary of patient demographics, clinical diagnoses, surgical approaches, and postoperative outcomes is presented in Table 1.

**Table 1:** Summary of patient cases undergoing minimally invasive cardiac surgery via periareolar approach

Case Study	Age	Gender	Diagnosis	ASA Classification	Surgical Approach	Cross Clamp Time (min)	ICU Stay (days)	Total Hospital Stay (days)	Scar Size (cm)	Scar Pigmentation	Numbness Duration (weeks)
1	29	Female	Mitral Valve Prolapse, Severe Mitral Valve Regurgitation	3	Periareolar Incision	103	1	5	3	None	2
2	17	Female	SV-ASD, PAPVD	3	Periareolar Incision	164	1	4	2.8	Minor Hypopigmentation	0
3	35	Female	Left Atrial Myxoma	3	Periareolar Incision	92	1	6	3.5	Mild Hyperpigmentation	4
4	42	Female	Large Secundum ASD	3	Periareolar Incision	100	1	5	5	Slight Hypopigmentation	0
5	57	Female	Secundum ASD, Cor Triatriatum sinister	3	Periareolar Incision	173	1	6	3	None	0

ASA: American Society of Anesthesiologists, SV-ASD: Superior Sinus Venosus Atrial Septal Defect, PAPVD: Partial Anomalous Pulmonary Venous Drainage, ICU: Intensive Care Unit, ASD: Atrial Septal Defect, min: minutes, cm: centimeters

## Discussion

This study provides valuable insights into the effectiveness of the periareolar incision technique in cardiac surgery for eligible patients. It emphasizes the importance of understanding the advantages, disadvantages, indications, and potential complications associated with different surgical approaches. By staying updated with new techniques and innovations, we can significantly improve healthcare outcomes and enhance the overall quality of life for patients undergoing cardiac surgery. Additionally, we have provided a narrative review of previous publications in Table 2.

**Table 2:** Summary of previous publications on minimally invasive cardiac surgery approaches, including the Periareolar incision technique

Study	Objective	Study Design	Sample size	Key findings
Van Praet KM, et al. [6]	This report emphasizes the authors’ personal experience utilizing a periareolar endoscopic approach, which prioritizes achieving excellent cosmetic outcomes while also ensuring optimal clinical results.	Retrospective study	109 Male patients	• The periareolar endoscopic approach in minimally invasive cardiac surgery (MICS) is a safe and aesthetically pleasing option. • It allows for mitral valve repair, replacement, and concomitant surgeries. • Various scar assessment scales consistently demonstrate satisfactory functional and cosmetic outcomes with this technique.
Bozso SJ, et al. [9]	The objective of this case report is to describe the successful minimally invasive, endoscopic repair of an unroofed coronary sinus atrial septal defect in a patient. The surgical procedure involved utilizing autologous pericardial baffle reconstruction to reconstruct the coronary sinus roof and closing the interatrial communication through a periareolar approach.	Case report	One patient	• The authors suggest that both simple and complex atrial septal defects (ASD) can be successfully repaired using minithoracotomy techniques. • They propose that the periareolar approach provides young female patients with an aesthetically pleasing alternative for minimally invasive surgery.
Poffo R, et al. [10]	The authors argue that utilizing the periareolar approach offers convenient access to cardiac structures. This is attributed to the positioning of the nipple-areolar complex at the advantageous 4th intercostal space, enabling direct entry to the heart.	Retrospective study	214 patients	• Periareolar access has shown to be a safe and effective method for treating various cardiac conditions. • This approach is known for delivering excellent aesthetic and functional outcomes, with low rates of complications in long-term follow-up. • In video-assisted procedures, particularly for female patients, periareolar access is the preferred approach.
Durdu MS, et al. [8]	The authors aimed to evaluate the surgical outcomes and gauge the level of cosmetic satisfaction linked to periareolar and submammary incisions in cardiac surgery.	Prospective cohort	94 female patients, 62 patients	• The study findings indicated that the periareolar approach showed superior aesthetic outcomes and improved healing. • Specifically, female patients experienced smaller scar sizes when the periareolar approach was utilized.
Baker RY, et al. [11]	The objective of this study was to evaluate the utilization of inframammary incision for minimally invasive cardiac surgery in patients who have breast implants.	Case series	5 Female patients	• The study findings suggest that performing minimally invasive cardiac surgery (MICS) with an inframammary incision is a safe approach for patients with breast implants.
Oliveira KAS, et al. [12] 2022	The objective of this study is to compare the in-hospital outcomes of patients who underwent video-assisted minimally invasive mitral valve repair using two distinct approaches: right minithoracotomy and periareolar access.	Retrospective study	37 patients.	• The study findings revealed a significant difference in the time to extubation between the right minithoracotomy group and the periareolar access group, with mean times of 4.85 h and 5.62 h, respectively (*P* = 0.04).

While the primary objective of cardiac surgery is to save lives, it is crucial to recognize the psychological, aesthetic, and mental well-being of patients as equally significant. Chest scarring has been shown to impact patients’ self-esteem and self-confidence, as well as various aspects of their lives such as career choices, success, relationships, and recreational activities [13]. This understanding has driven the development of new techniques in the surgical field that not only address the primary issue but also offer additional advantages. One such innovative technique is minimally invasive cardiac surgery, which reaps the benefits of the periareolar incision approach. Both patients and surgeons consistently report highly satisfactory results with this approach. The periareolar incision, a well-established technique in aesthetic and reconstructive surgery, has demonstrated minimal complications and excellent aesthetic outcomes [14, 15]. This approach greatly facilitates wound care, reduces complications for surgeons, and promotes faster healing, improved cosmetic outcomes, and fewer complications for patients. Furthermore, this approach has shown that it can be utilized for various cardiac diseases. As this approach provides perpendicular visualization of the surgical field which aids in the repair of different structural heart disease pathologies. On the other hand, angled visualization might be necessary in mitral valve procedures which can be easily achieved using an endoscopic camera. However, it is important to acknowledge that the need for a standby plastic surgery team in the hospital is a limitation of this technique. While our study focuses on the periareolar minimally invasive approach, it is important to consider alternative techniques that have been described in the literature. One such technique is the tumescent local anesthesia approach for breast augmentation in transgender patients, as reported by Tettamanzi et al. [16]. This technique offers potential advantages, such as reduced postoperative pain and faster recovery. However, the periareolar approach provides direct access to the cardiac structures without the need for extensive dissection, which may be beneficial in certain cases. Future studies should directly compare these approaches to determine their relative benefits and limitations in the context of minimally invasive cardiac surgery.

Studying these emerging techniques requires dedicated efforts, and this study represents the first investigational description and evaluation of the periareolar incision approach in Saudi Arabia. Patient data were meticulously documented, evaluated, and recorded to minimize potential recall bias. Nonetheless, limitations are inherent in any study. While we believe our sample size is adequate for a case series study, a larger sample size would undoubtedly provide more robust results. Our study design primarily focuses on describing and documenting patients’ experiences from initial presentation to final follow-up, thereby capturing short-term patient experiences and lacking long-term pre- and post-intervention data. This study predominantly focuses on qualitative variables, with limited coverage of quantitative variables. We are optimistic that this study will serve as a foundation for future research projects on the periareolar incision technique in our region and beyond. We strongly encourage enthusiastic researchers to explore this topic from various perspectives, as it is crucial for improving healthcare and enhancing the quality of life for patients who undergo minimally invasive cardiac surgery with a periareolar incision. Key areas for further investigation include patient selection, comparative analysis of aesthetic outcomes with different approaches, periareolar scar revision, and the management of unwanted cosmetic results. Additionally, it is important to examine the periareolar incision approach from the surgeon’s perspective, assessing its efficacy in providing optimal visualization, facilitating surgical procedures, and enabling comfortable management of unexpected complications or mistakes. In addition to the periareolar approach, other minimally invasive techniques have been described in the literature. Baker et al. [11] and Durdu et al. [8] reported the successful use of the inframammary approach for minimally invasive cardiac surgery, with good outcomes and patient satisfaction. These studies further support the notion that minimally invasive approaches can provide both functional and aesthetic benefits for patients undergoing cardiac surgery. Moreover, many women have previously undergone breast augmentation or biopsies, and these existing scars could potentially be utilized to access the chest cavity and heart, thus minimizing the need for additional incisions [11]. As technology and endoscopic techniques continue to advance, it is essential for surgeons to consider not only the functional aspects of the procedure but also the aesthetic outcomes that can have long-lasting effects on patients’ psychological well-being. By prioritizing both functional and aesthetic goals, we can improve overall patient satisfaction and quality of life. One notable limitation of our study is the absence of patient-reported outcome measures, such as the BREAST-Q questionnaire, which would have provided valuable insights into patient satisfaction and aesthetic outcomes. The BREAST-Q is a validated tool that comprehensively assesses patients’ post-surgical well-being, including satisfaction with breasts, psychological well-being, physical well-being, and sexual well-being [17]. The inclusion of this questionnaire would have strengthened our evaluation of the periareolar minimally invasive approach and its impact on patient-reported outcomes. Future studies should prioritize the incorporation of the BREAST-Q to address this limitation and enable a more robust assessment of patient satisfaction and aesthetic outcomes associated with this technique.

## Conclusion

The periareolar incision has emerged as a highly promising approach for MICS, offering numerous advantages while presenting minimal disadvantages. Our study findings support the effectiveness of the periareolar incision approach, with the majority of patients expressing high satisfaction and experiencing no complications related to the wound or cosmetic outcomes. The observed improvements in aesthetic, psychological, and physical outcomes highlight the potential of this approach in enhancing patient experiences and overall surgical outcomes. However, it is important to acknowledge that further extensive research is necessary to establish the periareolar incision approach as a standard of care for eligible surgical patients. Future studies should focus on larger patient cohorts and comparative analyses to validate the benefits of this approach against alternative techniques. Long-term follow-up assessments are needed to evaluate the durability of aesthetic outcomes and potential late complications. Additionally, multi-center studies and meta-analyses will contribute to a more comprehensive understanding of the periareolar incision technique’s efficacy and safety in diverse patient populations. By conducting rigorous research and accumulating substantial evidence, we can establish the periareolar incision approach as a preferred option in MICS procedures, ensuring improved surgical outcomes and patient satisfaction.

## Data Availability

The data are available upon request from the corresponding author.

## Abbreviations

MIMVr: Minimally invasive mitral valve repair
MIMVR: Minimally invasive mitral valve replacement
MICS: Minimally invasive cardiac surgery
HD: High definition
TEE: Transesophageal echocardiography
CPB: Cardiopulmonary bypass
ICU: Intensive care unit
ASA: American Society of Anesthesiologists
SV: ASD-Superior sinus venosus atrial septal defect
PAPVD: Partial anomalous pulmonary venous drainage
SVC: Superior vena cava
ASD: Atrial septal defect
